# The pathological mechanisms of circRNAs in mediating intervertebral disc degeneration

**DOI:** 10.1016/j.ncrna.2023.09.004

**Published:** 2023-09-18

**Authors:** Yongjin Li, Suzhe Zhou, Xinli Hu, Shibao Lu

**Affiliations:** a Department of Orthopedics, Xuanwu Hospital, Capital Medical University, No. 45 Changchun Street, Xicheng District, Beijing, China; b National Clinical Research Center for Geriatric Diseases, Beijing, China; c Department of Orthopedics, Anhui No 2 Provincial People's Hospital, Hefei, China

**Keywords:** Intervertebral disc degeneration, Nucleus pulposus cells, Circular RNAs, Extracellular matrix, Inflammatory response

## Abstract

Lower back pain (LBP) is a worldwide health problem associated with significant economic and social burden. Intervertebral disc degeneration (IVDD) is a leading cause of LBP. Several studies show that the death of nucleus pulposus cells (NPCs), abnormal metabolism of the extracellular matrix (ECM), and inflammatory response are the key mechanisms behind the pathogenesis of IVDD. Circular RNAs (circRNAs) are key regulators of gene expression and play a significant role in regulating NPCs death, ECM homeostasis, and inflammatory response by acting as microRNAs (miRNAs) sponges in IVDD. However, the regulatory role of circRNAs in mediating IVDD remains unknown. This review comprehensively describes the normal anatomic structure and function of IVD, the pathogenesis of IVDD, the characteristics, synthesis, mechanisms, and function of circRNAs. Moreover, we highlighted the 23 circRNAs that mediate ECM metabolism, 16 circRNAs that mediate NPCs apoptosis, circ_0004354 and circ_0040039 that mediate NPCs pyroptosis, and 5 circRNAs that mediate inflammatory response in IVDD. In addition, this review presents suggestions for future studies, such as the need for further investigation on ferroptosis-related circRNAs in IVDD. This review could provide novel insights into the pathogenesis and treatment of IVDD.

## 1 Introduction

Low back pain (LBP) is a common worldwide health problem in adults, associated with a huge economic and social burden to patients, families, and the society [1]. A previous systematic analysis conducted in 2019 on the global burden of 369 diseases in 204 countries revealed that LBP was the fourth cause of disability among people aged 25–74 years [2]. The Lancet series call for worldwide recognition of LBP. Furthermore, the prevention, diagnosis, treatment, and rehabilitation of patients with LBP is associated with various challenges [[2], [3], [4], [5], [6]]. Intervertebral disc degeneration (IVDD) is reported to be one of the main causes of LBP, with about 40% of LBP cases attributed to intervertebral disc degeneration (IVDD).

Intervertebral disc degeneration can be caused by various internal factors such as genetics and aging and external factors such as infections, trauma, and smoking. Genetic factors account for 34–61% of IVDD cases [4,[7], [8], [9]]. The nucleus pulposus (NP) tissue is located at the center of the intervertebral disc (IVD) and is mainly composed of nucleus pulposus cells (NPCs) and extracellular matrix (ECM). The NP is a gel-like substance composed of water, proteoglycans, elastin fibers, and proteins. The NP distributes hydraulic pressure throughout the intervertebral disc. Degeneration of the intervertebral disc usually starts from the nucleus pulposus. The NP comprises nucleus pulposus cells (NPCs) and an extracellular matrix (ECM). Furthermore, the physiological function of the nucleus pulposus depends on the homeostasis of its microenvironment and inflammatory response.

The NP lacks blood supply and self-repair ability. Disc degeneration could occur due to inflammation, ischemia, hypoxia, and acidic microenvironment [10]. The current management of IVDD aims to alleviate symptoms but does not reverse the degeneration. Moreover, surgery impairs the integrity of intervertebral discs, leading to accelerated degeneration, recurrence, and adjacent segment degeneration [[11], [12], [13]]. Gene therapy targeting the nucleus pulposus could block or reverse the pathological process of IVDD, thus offering a promising effective therapeutic strategy for IVDD [6,14].

Circular RNAs (circRNAs) are a major type of regulatory non-coding RNA, which can mediate the occurrence and development of IVDD by sponging microRNAs (miRNAs) and are involved in posttranscriptional gene regulation. circRNAs could be exploited as novel diagnostic and prognostic biomarkers. This review provides an update on the regulatory function of circRNAs in the degeneration of the nucleus pulposus. This review aims to provide a theoretical basis for understanding the potential therapeutic targets for developing novel therapies for IVDD.

### 1.1 The normal anatomic structure and function of IVD

The human spine comprises 23 vertebrae, including 6 cervical vertebrae, 12 thoracic vertebrae, and 5 lumbar vertebrae, accounting for about a quarter of the total height of the spine [15]. Each intervertebral disc can bear complex mechanical loads while maintaining spinal flexibility, including axial bending, compression, and rotation [16]. The intervertebral disc is a fibrocartilage located between two adjacent vertebrae, and is an important functional unit of the spine. It consists of a central gelatinous core called the nucleus pulposus, annulus fibrosus (AF) surrounding the nucleus pulposus, and the cartilage endplate that connects adjacent vertebrae [17]. The intervertebral disc is an avascular organ whereby blood vessels end at the cartilage endplate and do not cross through the nucleus pulposus or the inner layer of annulus fibrosus. Therefore, the nucleus pulposus lacks blood supply and exists in a hypoxic-ischemic microenvironment. However, oxygen and nutrients enter into the nucleus pulposus and annulus fibrosus through the cartilage endplate [18]. The cartilage endplate contains channels responsible for discharging metabolic wastes from the intervertebral disc [19,20].

The extracellular matrix (ECM) is a non-cellular, complex, and highly dynamic structure, that regulates cell function and promotes cell communication. The ECM plays an important role in maintaining the integrity and biomechanical function of intervertebral discs. In addition, the ECM can selectively bind to growth factors related to tissue morphogenesis, homeostasis, and repair [21]. The ECM of the nucleus pulposus is mainly composed of aggregate proteoglycans (ACAN) and collagen type II (COL2) in a ratio of 27:1. with COL2 accounting for about 20% of the dry weight of the nucleus pulposus. On the other hand, ACAN is the main proteoglycan of the nucleus pulposus, accounting for about 50% of the wet weight of the nucleus pulposus [10]. The proteoglycans are rich in chondroitin sulfate and are aggregated with hyaluronic acid, which helps maintain osmolar pressure in the nucleus polposus and helps the disc act as a shock absorber [10,22,23].

Previous studies reveal that ACAN is important in regulating the growth of nerves and blood vessels in the intervertebral disc [[22], [23], [24]]. For example, Stefanakis et al. [24] showed that IVD degradation and depletion of ACAN were associated with an influx of blood vessels and nerves. COL2 forms the collagen fiber network, which holds proteoglycans and provides the intervertebral disc with tensile strength [25]. Factors hindering the diffusion of oxygen and nutrients into the vertebral disc or discharge of metabolic wastes could lead to the reduction of oxygen tension in the intervertebral disc and anaerobic metabolism, thus leading to increased production of lactic acid and decreased PH. Consequently, this could affect the metabolic and biosynthetic function of NPCs thus impairing ECM synthesis and decomposition.

### 1.2 The pathogenesis of IVDD

**Fig. 1:**
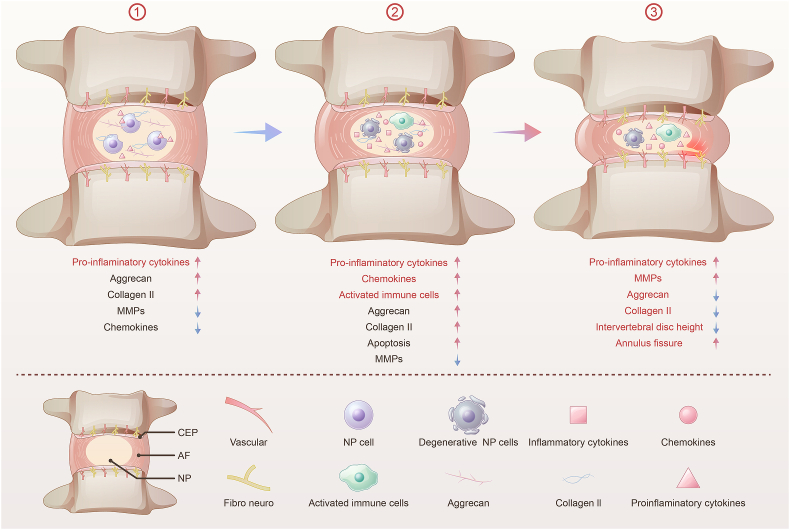
The pathological process of IVDD. (A) Secretion of proinflammatory factor by nucleus pulposus cells; (B) Apoptosis of nucleus pulposus cells and inflammatory response; (C) ECM degradation caused by MMPs.

The pathological process of IVDD is divided into three stages (Fig. 1): ① Stage 1 involves the secretion of proinflammatory mediators by nucleus pulposus cells (NPCs) due to various factors such as heredity, aging, infections, trauma, and smoking, and the high content of ECM and the low content of matrix metalloproteinases (MMPs), chemokines in intervertebral discs; ② The released proinflammatory factors cause apoptosis and pyroptosis of NPCs and further release of inflammatory factors; NPCs also secrete chemokines, which cause the activation and infiltration of CD4+and CD8+T cells, M1 macrophages, mast cells, neutrophils, and other immune cells, which also release more cytokines and chemokines. These cytokines and chemokines recruit more immune cells into the intervertebral disc, which secrete proinflammatory cytokines leading to an inflammatory cascade. In this stage, there is still a high ECM in the intervertebral discs.③ The proinflammatory factors and the inflammatory cascade promote the expression of matrix metalloproteinases (MMPs), leading to ECM degradation、reduced intervertebral discs height, and annulus fissure, thus causing IVDD [[4], [5], [6],^26^]. Progression of IVDD is associated with decreased water content of the nucleus pulposus tissue, decreased diffusion of oxygen and nutrients, and decreased discharge of metabolic wastes from the intervertebral disc. These factors worsen the hypoxic environment in the intervertebral disc and lead to anaerobic metabolism, thus increasing lactic acid production and accumulation and decreasing the pH value of the microenvironment, further promoting NPCs death and ECM degradation [10,27]. The altered homeostasis, ECM degradation, and inflammation of intervertebral disc lead to changes in the morphology (the height of the intervertebral disc decreases) [28], growth of microvessels and nerve fibers into the intervertebral disc [4,23,24,26,29]), decrease water content in the intervertebral disc [30]), lead to loss of flexibility and elasticity [31], and cause biochemical alterations [[4], [5], [6],^26^] of the intervertebral disc. Therefore, the key pathogenic mechanisms behind IVDD include the increased apoptosis of NPCs in the microenvironment of intervertebral discs [32], the enhanced inflammatory response [[4], [5], [6],^26^], and increased ECM degradation [10,33].

#### 1.2.1 Characteristics, synthesis, mechanism, and function of CircRNA

Non-coding RNAs (ncRNA) mainly include circular RNAs (circRNA), long non-coding RNAs (lncRNAs), and microRNAs (miRNAs). Genes of non-coding RNAs are transcribed in a similar manner to the protein-coding genes. However, ncRNAs are not translated into proteins^[^ [34]^].^ CircRNAs are important posttranscriptional regulators, whose 5′ and 3′ ends are joined together to form closed circular loop structures. CircRNAs are highly stable and are not easily degraded by exonucleases [[35], [36], [37]]. CircRNAs show tissue expression specificity and developmental stage specificity [[38], [39]]. Abnormal expression of circRNAs is associated with various human diseases. Since circRNAs are highly stable, they could be exploited as potential biomarkers for the diagnosis and prognosis of several diseases, such as spinal diseases [[40], [41], [42], [43]], neurodegenerative diseases [44,45], cardiovascular diseases [46], autoimmune diseases [47], and cancers [[48], [49], [50]].

Seven types of circRNAs have been identified based on their splice positions in the genome [42], including ecircRNA (from exonic regions), ciRNA (from intronic regions), eiciRNA (from both exonic and intronic regions), read through circRNA (rt circRNA) formed by cyclization of two exons of two different genes, tricRNA formed by pre-tRNA intron splicing [51], fusion gene derived f-circRNA [52], and mecciRNA encoded by the mitochondrial gene [53]. Among them, ecircRNA is the most common type, which is spliced from at least one exon of a single gene. ecircRNAs are usually located in the cytoplasm, where they function as miRNA sponges [42].

Most circRNAs are formed in the nucleus through reverse splicing of pre-mRNA. In addition, circRNAs play various biological roles within the cell nucleus and cytoplasm [[35], [36], [37]]. CiRNAs and eiciRNAs are predominantly located in the nucleus and are involved in the regulation of gene transcription [50,54], variable splicing [55], and parental gene expression [56]. The ecircRNAs are mainly located in the cytoplasm. ecircRNAs competitively bind to miRNAs [57], proteins [58], regulate protein translation [59], and encode proteins [[60], [61], [62]].

## 2 Regulatory role of circRNAs in degeneration of nucleus pulposus

### 2.1 ECM metabolism

**Fig. 2:**
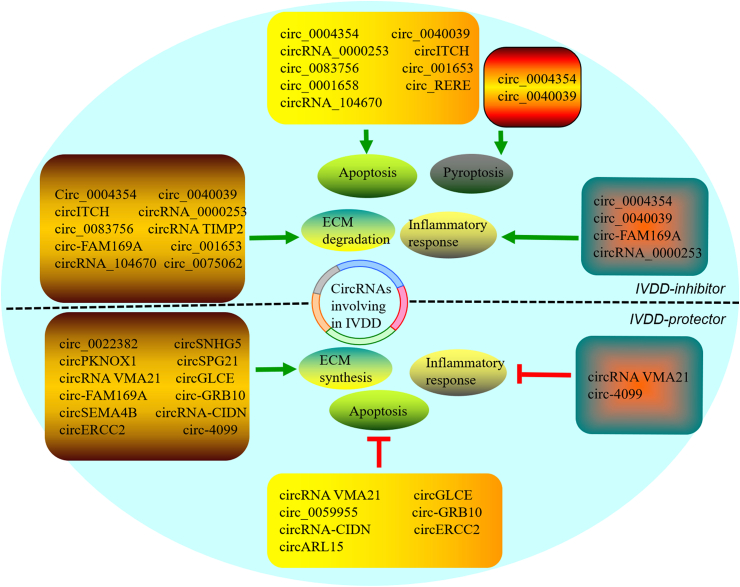
Different circRNAs act as protector or inhibitor in IVDD by regulating ECM metabolism, apoptosis, pyroptosis, and inflammatory response. In the upper half of the ellipse, 10 circRNAs promote ECM degradation; 9 circRNAs promote cells apoptosis; 2 circRNAs promote cells pyroptosis; 4 circRNAs promote inflammatory response; thus, these IVDD-related circRNAs were regarded as IVDD-inhibitor. In the lower half of the ellipse, 12 circRNAs promote ECM synthesis; 7 circRNAs inhibit cells apoptosis; 2 circRNAs inhibit inflammatory response; thus, these IVDD-related circRNAs were regarded as IVDD-protector.

Previous studies have shown that circRNAs play an important role in IVDD by regulating apoptosis, pyroptosis, ferroptosis, and necrosis of NPCs, ECM degradation, and inflammatory response [42,43]. To date, 25 circRNAs associated with IVDD have been reported, including 12 circRNAs that promote IVDD progression and 13 circRNAs that inhibit IVDD progression. Furthermore, 23 circRNAs are reported to be involved in the regulation of ECM metabolism, including 10 circRNAs which promote ECM catabolism (Circ_0004354 [63], circ_0040039 [63], circITCH [64], Circ_0083756 [65], Circ-FAM169A [66], circRNA_0000253 [67], circRNA TIMP2 [68], cirC_001653 [69], circRNA_104670 [70], and circ_0075062 [71]) and 12 circRNAs that promote ECM synthesis (circ_0022382 [72], circSPG21 [73], circSNHG5 [74], circPKNOX1 [75], circGLCE [76], circRNA VMA21 [77], Circ-grb10 [78,79], circRNA-CIDN [80], circERCC2 [81], circSEMA4B [82], circ-4099 [83], and circ-FAM169A [84]) (Table 1 and Fig. 2). However, the role of circ-FAM169A in ECM metabolism is still controversial.

**Table 1:** The IVDD-related circRNAs involved in regulating ECM metabolism, apoptosis, pyroptosis, and inflammatory response.

Author, year	circRNA	Expression	Targeted pathway	Functions
Li et al.*,* 2022 [63]	circ_0004354 circ_0040039	Upregulated	miR-345-3p-FAF1/TP73	Pyroptosis Apoptosis Inflammatory response ECM degradation
Zhang et al.*,* 2021 [64]	circITCH	Upregulated	miR-17-5p-SOX4	Apoptosis ECM degradation
Du et al.*,* 2022 [65]	circ_0083756	Upregulated	miR-558-TREM1	ECM degradation
Guo et al.*,* 2020 [66]	circ-FAM169A	Upregulated	miR-583-BTRC	ECM degradation
Song et al.*,* 2020 [67]	circRNA_0000253	Upregulated in exosome	miRNA-141-5p-SIRT1	Apoptosis ECM degradation
Guo et al.*,* 2020 [68]	circRNA TIMP2	Upregulated	miR-185-5p-MMP2	ECM degradation
Cui et al.*,* 2020 [60]	circ_001653	Upregulated	miR-486-3p- CEMIP	ECM degradation
Song et al.*,* 2018 [70]	circRNA_104670	Upregulated	miR-17-3p- MMP2	Apoptosis ECM degradation
Chang et al.*,* 2021 [71]	circ_0075062	Upregulated	Unknown	ECM degradation
Hu et al.*,* 2022 [72]	circ_0022382	Downregulated	miR-4726-5p-TGFβ3	ECM synthesis
Huang et al.*,* 2021 [73]	circSPG21	Downregulated	miR-1197-ATP1B3	ECM synthesis
Zhang et al.*,* 2021 [74]	circSNHG5	Downregulated	miR-495-3p-CITED2	ECM synthesis
Huang et al.*,* 2021 [75]	circPKNOX1	Downregulated	miR-370-3p-KIAA0355	ECM synthesis
Chen et al.*,* 2020 [76]	circGLCE	Downregulated	miR-587-STAP1	ECM synthesis Apoptosis
Cheng et al.*,* 2018 [77]	circRNA VMA21	Downregulated	miR-200c-XIAP	ECM synthesis Apoptosis Inflammatory response
Guo et al.*,* 2018 [78]	circ-GRB10	Downregulated	miR-328-5p-ERBB2	ECM synthesis Apoptosis
Guo et al.*,* 2020 [79]	circ-GRB10	Downregulated	miR-141-3p-FUS	ECM synthesis
Xiang et al.*,* 2020 [80]	circRNA-CIDN	Downregulated	miR-34a-5p-SIRT1	Apoptosis ECM synthesis
Xie et al.*,* 2019 [81]	circERCC2	Downregulated	miR-182-5p-SIRT1	ECM synthesis Apoptosis
Wang et al.*,* 2018 [82]	circSEMA4B	Downregulated	miR-431-SFRP1/GSK-3β	ECM synthesis
Wang et al.*,* 2018 [83]	circ-4099	Upregulated	miR-616-5p-Sox9	Inflammatory response ECM synthesis
Li et al.*,* 2020 [84]	circ-FAM169A	Upregulated	miR-583-Sox9	ECM synthesis Apoptosis
Wang et al.*,* 2021 [85]	circ_RERE	Upregulated	miR-299-5p-Galectin-3	Apoptosis
Meng et al.*,* 2021 [86]	circ_0001658	Upregulated	miR-181c-5p-FAS	Apoptosis
Wang et al.*,* 2021 [87]	circARL15	Downregulated	miR-431-5p-DISC1	Apoptosis
Kong et al.*,* 2020 [88]	circ_0059955	Downregulated	Unknown	Apoptosis

According to Li et al. [63], circ_0004354 may compete with circ_0040039 to promote pyroptosis, apoptosis, inflammation, and inhibition of NPC cell proliferation and ECM degradation by targeting the miR-345-3P-FAF1/TP73 axis. In addition, circITCH acts as a sponge for miR-17–5p to derepress SOX4, thus promoting the Wnt/β-catenin signaling axis to accelerate apoptosis of NPCs, growth inhibition, and ECM degradation [64]. Furthermore, Du et al. [65] showed significantly increased expression of circ_0083756 in degenerative nucleus pulposus tissues, which promoted NPCs apoptosis, growth inhibition, and ECM degradation by targeting the miR-558-TREM1 pathway. Guo et al. [66] revealed that the circ-FAM169A-miR-583/BTRC signaling axis regulates the NF-κB pathway-induced IL-1β and TNF-α production and ECM degradation. Furthermore, Song et al. [67] demonstrated that circRNA_0000253 promoted cell apoptosis and ECM degradation by inhibiting miRNA-141–5P and down-regulating SIRT1. On the other hand, circ-TIMP2 promotes ECM degradation through the miR-185-5p-MMP2 signaling pathway [68]. Cui and Zhang [69] reported that the circ_001653-miR-486-3p-CEMIP signaling axis could promote NPCs apoptosis and enhance ECM catabolism. CircRNA_104670 was shown to promote the expression of MMP-2, enhance NPCs apoptosis, growth inhibition, and ECM degradation. In addition, circRNA_104670 promotes IVDD by inhibiting miR-17–3p [70]. Knockdown of circ_0075062 in glucose-deficient NPCs was shown to inhibit ECM degradation [71].

Among the 12 circRNAs that promote ECM anabolism, circ-4099 was shown to be significantly up-regulated in IVDD, while the other 11 circRNAs were significantly down-regulated in IVDD.

The circ_0022382-miR-4726-5p-transforming growth factor-3 (TGF-β3) axis plays a key role in the synthesis and catabolism of ECM in endplate chondrocytes [72]. Huang et al. reported that circSPG21 could regulate the mir-1197-ATP1B3 pathway and promote ECM synthesis [73]. In addition, the circSNHG5-miR-495–3p -CITED2 signaling axis plays an important role in regulating chondrocyte proliferation and ECM anabolism [74]. Knockdown of CircPKNOX1 promotes ECM degradation through the mir-370-3P-KIAA0355 pathway [75]. Chen et al. [76] showed that overexpression of circGLCE could alleviate the mir-587-induced inhibition of STAP1, inhibit the expression of ECM degradation enzymes, and inhibit apoptosis of NPCs. CircVMA21 acts as a sponge for miR-200c and could alleviate NPCs apoptosis and ECM degradation by inhibiting the miR-200c-XIAP (X-linked inhibitor of apoptosis protein) pathway. A previous study revealed that injection of circVMA21 into the caudal intervertebral disc of rat models resulted in a delay of IVDD [77]. Guo et al. [78,79] reported that overexpression of circ-GRB10 inhibited miR-328–5p and increased the expression of ERBB2 through the mTOR pathway, thereby alleviating apoptosis of NPCs. The miR-141-3p-FUS pathway is a key pathway in the synthesis of circ-GRB10 in NPCs. Furthermore, the circ-GRB10-miR-328-5p-ERBB2 pathway regulates the phosphorylation of ERK1/2 and enhances the production of miR-141–3p in NPCs, forming a positive feedback loop. A previous study showed that overexpression of circRNA-CIDN could rescue compression-induced NPC apoptosis and ECM degradation through sponging miR-34a-5p that inhibited SIRT1 expression [80]. Circ-ERCC2 could remove the inhibition of mir-182–5p on SIRT1 by sponging miR-182–5p, thus activating mitophagy, reducing NPCs apoptosis, and inhibiting IVDD [81]. Wang et al. [82] reported that the circSEMA4B-miR-431-GSK-3β/SFRP1 signaling axis attenuates IL-1β-induced NPCs senescence, ECM, and ACAN degradation by regulating Wnt signaling. Furthermore, circ-4099 was shown to relieve the inhibition of SOX9 by sponging miR-616–5p, thus promoting ECM synthesis and inhibiting the release of prostaglandin E2, TNF-α, and IL-1β [83]. Li et al. [84] showed that circ-FAM169A could promote ECM synthesis through the miR-583-SOX9 pathway.

### 2.2 Nucleus pulposus cell death

Cell death is important in organism development, host defense against pathogens, and tissue homeostasis. Cell death is divided into accidental cell death and regulated cell death (RCD). Accidental cell death is an uncontrolled biological process, while RCD involves tightly regulated signaling cascades and molecularly defined effector mechanisms [85]. Several types of RCD have been identified. Tang et al. [85] reported that RCD was divided into 12 types, including apoptosis, pyroptosis, ferroptosis, and necrotizing apoptosis. Previous studies showed that the death of NPCs, resulting in decreased disc height and ECM synthesis, plays an important role in the occurrence and development of IVDD [32,86]. Apoptosis was the first type of RCD to be identified [85]. Sixteen circRNAs have been reported to regulate NPCs apoptosis, including nine circRNAs that were shown to promote NPCs apoptosis (circ_0004354 [63], circ_0040039 [63], circITCH [64], circ_0083756 [65], circRNA_0000253 [67], circ_001653 [69], circRNA_104670 [70], circ_RERE [87], and circ_0001658 [88]) and seven circRNAs that were shown to inhibit NPCs apoptosis (circGLCE [76], circRNA VMA21 [77], circ- GRB10 [78,79], circRNA-CIDN [80], circERCC2 [81], circARL15 [89], and circ_0059955 [90]) (Table 1 and Fig. 2). Wang et al. [87] reported that circ_RERE promoted hydrogen peroxide-induced apoptosis and autophagy in NPCs through the miR-299–5p/galectin-3 axis. Furthermore, circ_0001658 promotes NPCs apoptosis and inhibits cell proliferation by targeting the miR-181c-5p-FAS pathway [88]. CircARL15 acts as a miR-431–5p sponge to relieve the inhibition of DISC1, promote the proliferation of NPCs and inhibit the apoptosis of NPCs [89]. Kong et al. [90] demonstrated that the knockdown of circ_0059955 induced cell cycle arrest and promoted NPCs apoptosis by downregulating ITCH.

Pyroptosis is a type of regulated inflammatory cell death executed by Gasdermin family proteins, which form pores on the cell membrane. The activation of inflammatory CASPs promotes the secretion of IL-1β and IL-18 in pyroptosis [91]. Zhang et al. [92] showed significantly decreased expression of miR-410 in needle puncture-induced IVDD models. miR-410 is a key negative regulator of NPCs pyroptosis. Xu et al. [93] reported that miR-141 was significantly overexpressed in IVDD, which induced NPCs pyroptosis, ECM catabolism, and inflammation by increasing ROS production and stimulating the TXNIP-NLRP3 signaling axis. Interestingly, under TNF-α induction, circ_0004354 and circ_0040039 promoted the expression of cysteine aspartate proteinase 3 (CASP3) and GSDME to varying degrees, which participated in the regulation of NPCs pyroptosis and promoted the secretion of IL-1β [63] (Table 1 and Fig. 2).

Ferroptosis, an iron-dependent non-apoptotic and non-pyroptotic cell death, accompanied by lipid peroxidation and increased reactive oxygen species (ROS), was first reported by Dixon et al. [94]. Ferroptosis is related to the pathogenesis of IVDD. In addition, previous studies showed that inhibiting ferroptosis could be exploited as an effective strategy to delay IVDD [[95], [96], [97]]. GPX4 is an important marker of ferroptosis. A previous study showed decreased levels of GPX4 and ferritin heavy chain in a rat model of IVDD [95]. Sheng et al. [98] showed that the IL-6-miR-10a-5p-IL-6R signaling axis plays an essential role in regulating IVDD. Il-6 inhibits the expression of IL-6R by inhibiting miR-10A-5p, thereby inhibiting IL-6-induced ferroptosis. Previous studies showed that circRNAs mediate ferroptosis by regulating key proteins. In addition, circRNAs play an important role in various diseases, including cancer and traumatic brain injury [98,99]. However, circRNAs-mediated ferroptosis in IVDD has rarely been reported and needs further investigation.

### 2.3 Inflammatory reaction

The inflammatory reaction is caused by overexpression of inflammatory cytokines. Intervertebral disc degeneration is characterized by elevated levels of inflammatory cytokines, such as TNF-α, IL-1α, IL-1β, IL-6, IL-17, IL-18, and IFN-γ, and an inflammatory cascade, which promotes the production of matrix metalloproteinase and other catabolic factors that induce ECM degradation and enhance cell death. Impairment in the normal physiological function of the intervertebral disc may cause IVDD, painful nerve fibers, and growth of microvascular vessels into the intervertebral disc, leading to low back pain [[4], [5], [6],^26^,^93^,^100^]. Furthermore, available evidence reveals that dysregulation of circRNAs expression is associated with the production of inflammatory cytokines during IVDD. Five circRNAs have been reported to regulate the inflammatory response in IVDD, including four proinflammatory circRNAs (circ_0004354 [63], circ_0040039 [63], circ-FAM169A [66], and circRNA_0000253 [67]) and one anti-inflammatory circRNAs: circ-4099 [83] (Table 1 and Fig. 2). Cheng et al. showed that circRNA VMA21 promotes XIAP expression by binding miRNA-200C [77]. Increasing evidence reveals that XIAP can regulate the inflammatory response, while XIAP inhibition can promote the excessive secretion of TNF-α and IL-1β [101,102]. However, further studies are needed to investigate whether circRNA VMA21 regulates XIAP or mediates inflammation in IVDD.

## 3 Conclusion

Intervertebral disc degeneration is the main cause of low back pain, which affects the physical and mental health of patients. IVDD begins in the nucleus pulposus and is associated with various pathological mechanisms of NPCs, including apoptosis, pyroptosis, ferroptosis, inflammation, and ECM degradation. Recent studies showed that circRNAs regulate the expression of key proteins, including transcription factors related to IVDD, by acting as miRNA sponges, mediating NPCs death, inflammation, and ECM-related signaling pathways. In addition, circRNAs are involved in the pathological process of IVDD.

However, our understanding of circRNAs is still limited, and several questions remain unanswered. First, several circRNAs have been shown to play crucial roles in IVDD. However, studies have not revealed the key circRNAs involved in IVDD. Second, it is not clear whether different key circRNAs play different roles at different pathological stages of IVDD. Third, the upstream mediators involved in the dysregulation of the expression of most circRNAs in NPCs and their association with various risk factors of IVDD, such as genetics, aging, smoking, and trauma, remain largely unknown. In addition, it is not clear what factors affect the expression and function of circRNAs. Fourth, only a few studies report on the circRNAs that are dysregulated in other cell types associated with IVDD, such as cartilage endplate chondrocytes and annulus fibrosus cells. Fifth, it has not been reported whether circRNAs mediate IVDD progression through coding proteins, regulatory protein translation, parental gene transcription, or interaction with RNA-binding proteins. Therefore, further studies are needed to clarify this. Sixth, only a few studies report on the circrNA-circrNA interaction. Seventh, the use of serum circRNAs as diagnostic IVDD markers has not been reported. In the future, large-scale screening and validation of serum circRNAs related to diagnosis and prognosis should be carried out. Eighth, how circRNAs could be applied in the clinical setting to treat IVDD disease remains a huge challenge. Patients with early-to mid-stage IVDD may not present with severe symptoms. Therefore, there is a need to identify IVDD in the early stages to prevent IVDD progression and implement early interventions for the treatment of IVDD, thus improving patients' quality of life.

## Ethics approval and consent to participate

Not applicable.

## Patient consent for publication

Not applicable.

## Funding

This study was funded by National Natural Youth Cultivation Project of Xuanwu Hospital of Capital Medical University (QNPY2022022), and 10.13039/501100005024Beijing Postdoctoral Research Foundation (2023-zz-024).

## Author contributions

S.B.L. and Y.J.L. designed the experiments and wrote the article. X.L.H. collected the references. S.Z.Z. made the figure. S. Bfig.L. revised the manuscript. All authors approved the final version.

## Availability of data and material

The data that support the findings of this study are available from the corresponding author upon reasonable request.

## Declaration of competing interest

The authors declare no competing interests.
